# Superantigenic character of an insert unique to SARS-CoV-2 spike supported by skewed TCR repertoire in patients with hyperinflammation

**DOI:** 10.1073/pnas.2010722117

**Published:** 2020-09-28

**Authors:** Mary Hongying Cheng, She Zhang, Rebecca A. Porritt, Magali Noval Rivas, Lisa Paschold, Edith Willscher, Mascha Binder, Moshe Arditi, Ivet Bahar

**Affiliations:** ^a^Department of Computational and Systems Biology, School of Medicine, University of Pittsburgh, Pittsburgh, PA 15213;; ^b^Department of Pediatrics, Division of Pediatric Infectious Diseases and Immunology, Cedars-Sinai Medical Center, Los Angeles, CA 90048;; ^c^Biomedical Sciences, Infectious and Immunologic Diseases Research Center, Cedars-Sinai Medical Center, Los Angeles, CA 90048;; ^d^Department of Internal Medicine IV, Oncology/Hematology, Martin Luther University Halle-Wittenberg, 06120 Halle (Saale), Germany

**Keywords:** COVID-19, superantigen, SARS-CoV-2 spike, toxic shock syndrome, TCR binding

## Abstract

Multisystem Inflammatory Syndrome in Children (MIS-C) associated with COVID-19 is a newly recognized condition in children with recent severe acute respiratory syndrome coronavirus 2 (SARS-CoV-2) infection. These children and adult patients with severe hyperinflammation present with a constellation of symptoms that strongly resemble toxic shock syndrome, an escalation of the cytotoxic adaptive immune response triggered upon the binding of pathogenic superantigens to T cell receptors (TCRs) and/or major histocompatibility complex class II (MHCII) molecules. Here, using structure-based computational models, we demonstrate that the SARS-CoV-2 spike (S) glycoprotein exhibits a high-affinity motif for binding TCRs, and may form a ternary complex with MHCII. The binding epitope on S harbors a sequence motif unique to SARS-CoV-2 (not present in other SARS-related coronaviruses), which is highly similar in both sequence and structure to the bacterial superantigen staphylococcal enterotoxin B. This interaction between the virus and human T cells could be strengthened by a rare mutation (D839Y/N/E) from a European strain of SARS-CoV-2. Furthermore, the interfacial region includes selected residues from an intercellular adhesion molecule (ICAM)-like motif shared between the SARS viruses from the 2003 and 2019 pandemics. A neurotoxin-like sequence motif on the receptor-binding domain also exhibits a high tendency to bind TCRs. Analysis of the TCR repertoire in adult COVID-19 patients demonstrates that those with severe hyperinflammatory disease exhibit TCR skewing consistent with superantigen activation. These data suggest that SARS-CoV-2 S may act as a superantigen to trigger the development of MIS-C as well as cytokine storm in adult COVID-19 patients, with important implications for the development of therapeutic approaches.

## Significance

A hyperinflammatory syndrome reminiscent of toxic shock syndrome (TSS) is observed in severe COVID-19 patients, including children with Multisystem Inflammatory Syndrome in Children (MIS-C). TSS is typically caused by pathogenic superantigens stimulating excessive activation of the adaptive immune system. We show that SARS-CoV-2 spike contains sequence and structure motifs highly similar to those of a bacterial superantigen and may directly bind T cell receptors. We further report a skewed T cell receptor repertoire in COVID-19 patients with severe hyperinflammation, in support of such a superantigenic effect. Notably, the superantigen-like motif is not present in other SARS family coronaviruses, which may explain the unique potential for SARS-CoV-2 to cause both MIS-C and the cytokine storm observed in adult COVID-19.

Severe acute respiratory syndrome coronavirus 2 (SARS-CoV-2), the virus that causes COVID-19, is a betacoronavirus (β-CoV) closely related to SARS-CoV and Middle East Respiratory Syndrome (MERS)-CoV (1). COVID-19 can manifest in adults as a severe interstitial pneumonia with hyperinflammation, while severe respiratory manifestations are rare in children (2–4). Recently, however, multisystem inflammatory syndrome in children (MIS-C) has been observed in patients that either tested positive for COVID-19 (by PCR or serology) or had epidemiological links to COVID-19 (5–7). After initial reports in the United Kingdom (5), many cases have been reported in Europe (6, 7) and New York (Centers for Disease Control and Prevention). However, no such cases have been reported in China, Japan, or South Korea, which have also been severely impacted by the COVID-19 pandemic (European Centre for Disease Prevention and Control).

MIS-C manifests as persistent fever and hyperinflammation with multiorgan system involvement including cardiac, gastrointestinal (GI), renal, hematologic, dermatologic, and neurologic symptoms (5–7). These symptoms are highly reminiscent of toxic shock syndrome (TSS) (8, 9) (Table 1), rather than Kawasaki disease (KD), due to marked demographic, clinical, and laboratory differences (6). Indeed, a recent uncontrolled retrospective case study concluded that MIS-C is distinct from KD and KD shock syndrome (10). The similarities to TSS and the association of MIS-C with COVID-19 led us to hypothesize that SARS-CoV-2 may possess superantigenic fragments that induce an inflammatory cascade and may also contribute to the hyperinflammation and cytokine storm observed in severe adult COVID-19 patients (3, 4). The question we raised is, does SARS-CoV-2 spike (S) possess superantigenic fragments that could elicit such reactions upon binding proteins involved in the host cell cytotoxic adaptive immune response? Such a reaction was not observed in the SARS-CoV pandemic of 2003 (SARS1 hereafter). What is unique to SARS-CoV-2, and how might recent mutations in SARS-CoV-2 S have promoted such an increased virulence?

**Table 1.** Similarities between clinical and laboratory features of MIS-C and pediatric TSS

Clinical Features	MIS-C*	Pediatric TSS^†^
High fever	+	+
Skin rash	+	+
Conjunctivitis	+	+
Oral mucosal involvement	+	+
Myalgia	+	+
Hypotension	+	+
Myocardial involvement (dysfunction)	+	+
Gastro-intestinal symptoms (vomiting, diarrhea, abdominal pain)	+	+
Renal involvement	+	+
CNS symptoms, altered mental state	+	+
Headache	+	+
High C-reactive protein (CRP)	+	+
High ferritin	+	+
High IL-6	+	+
High D-dimers	+	+
High procalcitonin	+	+
Lymphopenia	+	+
Reduced platelet count	+	+
Increased neutrophil count	+	+
Increased aspartate amino transferase (AST) and alanine transaminase (ALT)	+	+
High pro b-type natriuretic peptide (pro-BNP)	+	NA
High troponin	+	NA
Isolation of TSS inducing bacteria (*Staphylococcus* or *Streptococcus*)	–	+

NA, not available; + represents association with reported cases; –, no association.

* Taken from refs. 5 to 7.

^†^ Taken from refs. 8, 9, 61, and 62.

TSS can be caused by two types of superantigens (SAgs): bacterial or viral. Bacterial SAgs have been broadly studied. They include proteins secreted by *Staphylococcus aureus* and *Streptococcus pyogenes* that stimulate massive production of inflammatory cytokines and toxic shock. Typical examples are TSS toxin 1, and staphylococcal enterotoxins B (SEB) and H (SEH). They are highly potent T cell activators that can bind to major histocompatibility complex (MHC) class II (MHCII) molecules and/or to T cell receptors (TCRs) of both CD4+ and CD8+ T cells. The ability of SAgs to bypass the antigen specificity of the TCRs results in broad activation of T cells and a cytokine storm, leading to toxic shock (11, 12). Notably, SAgs do not bind the major (antigenic) peptide-binding groove of MHCII, but instead bind other regions as well as the αβTCRs, directly. While early studies showed that bacterial SAgs activate T cells by binding to the β-chain of dimeric TCRs at their variable domain (V) (13–15), more-recent studies revealed that they can bind to either α- or β-chains, or both (16). The question is then, does SARS-CoV-2 S possess any superantigenic fragments/domains that could bind to TCRs?

Here, we used computational modeling to determine whether SARS-CoV-2 S possesses SAg-like fragments and activity. We demonstrate that a polybasic insert present in SARS-CoV-2 S, which is absent in the S glycoprotein of other SARS-related β-CoVs, mediates high-affinity, nonspecific binding to the TCR. Notably, a motif of ∼20 amino acids enclosing this insert unique to SARS-CoV-2 has sequence and structure features highly similar to those of the toxin SEB. Our analysis further indicates that a rare SARS-CoV-2 S mutation detected in a European strain may potentially enhance TCR binding. Moreover, analysis of a cohort of adult COVID-19 patients reveals that those with severe hyperinflammatory disease exhibit skewing of the TCR repertoire consistent with SAg activity. Therefore, these findings have important implications for the management and treatment of both MIS-C and COVID-19 patients with hyperinflammatory syndrome.

## Results and Discussion

### SARS-CoV-2 Spike Harbors a High-Affinity Site for TCR β-Chain Binding, which Contains An Insertion, P_681_RRA_684_, Unique to SARS2.

**Fig. 1.**
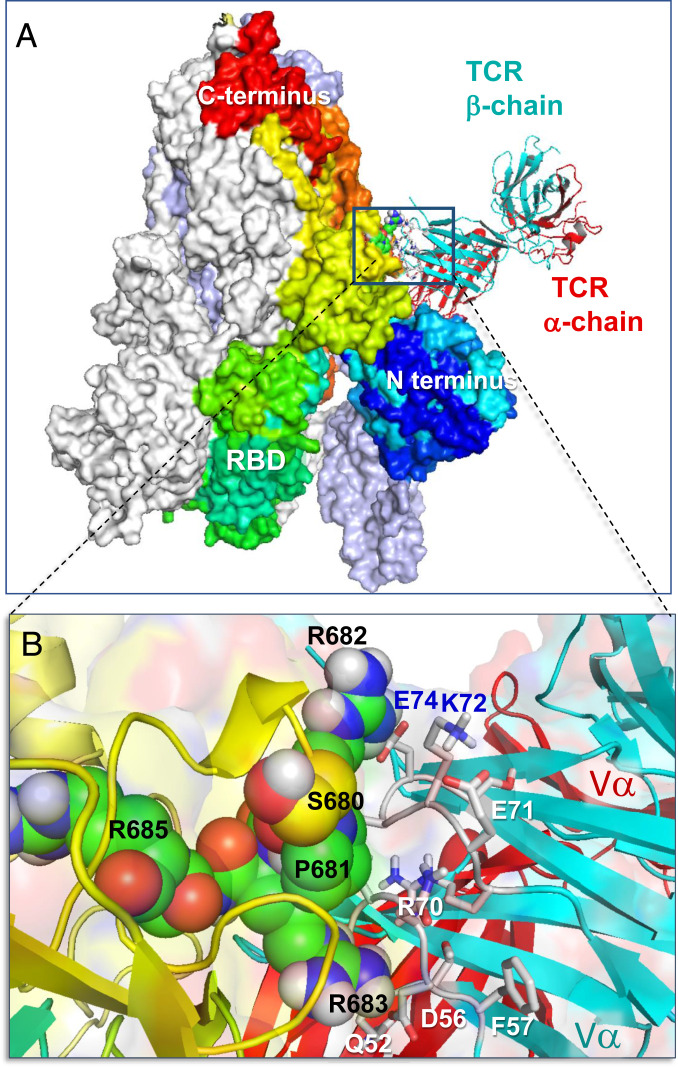
Binding of TCR to SARS-CoV-2 spike trimer near the “PRRA” insert. (*A*) Overall and (*B*) close-up views of the complex and interfacial interactions. In *A*, the spike monomers are colored white, ice blue/gray, and spectrally from blue (N-terminal domain) to red, all displayed in surface representation. The N and C termini and RBD of the spectrally colored monomer, which also binds the TCR, are labeled; for better visualization, the S trimer is oriented such that its RBDs are at the bottom. TCR α- and β-chains are in red and cyan ribbons. In *B*, the segment S_680_PPRAR_685_ including the PRRA insert and the highly conserved cleavage site R685 is shown in van der Waals representation (black labels); nearby CDR residues of the TCR Vβ domain are labeled in blue/white. See additional information in *SI Appendix*, Fig. S1.

We first examined whether SARS-CoV-2 S could bind to a TCR α- and/or β-chain. To this aim, we constructed a structural model for SARS-CoV-2 S based on the cryoelectron microscopy (cryo-EM) structure resolved for the S glycoprotein (17). We used then the X-ray structure of αβTCR resolved in a ternary complex with SEH and MHCII (16) to generate a series of structural models for possible SARS-CoV-2 S–TCR complex formation using ClusPro (18). Our simulations revealed two most probable TCR-binding sites on each monomer of S: one on the receptor-binding domain (RBD; residues R319 to K529), and the other near the S1/S2 cleavage site between the subunits S1 and S2. The former was also shared by SARS1 and MERS-CoV S, while the latter was unique and strongly preferred in SARS-CoV-2 S, as described in detail in *SI Appendix*, Figs. S1 and S2. Therefore, we focused on the latter, shown in Fig. 1*A*, as the most probable mechanism of complex formation that distinguishes SARS-CoV-2 S from other β-CoVs. A close-up view of the interface between S and TCRVβ (Fig. 1*B*) reveals strong interatomic interactions involving spike residues S680-R683, and Vβ residues Q52, D56, R70 to E74 (CDR2), and S96 to Q103 (CDR3).

**Fig. 2.**
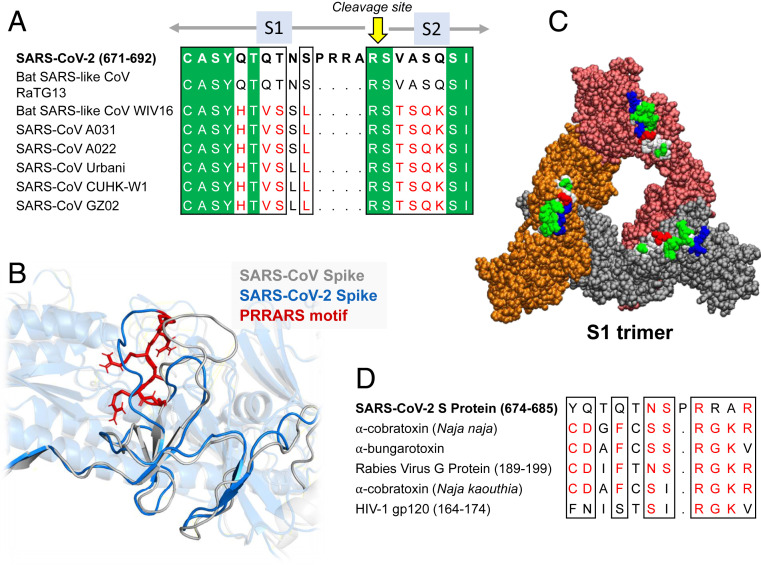
Sequence and structural properties of the insert PRRA. (*A* and *B*) SARS-CoV-2 encodes both a cleavage site and SAg-like motifs (20) near the insertion PRRA that distinguishes it from all SARS-related β-CoVs. (*A*) Sequence alignment of SARS-CoV-2 and multiple SARS-related strains (1) near the insertion PRRA. (*B*) Structural alignment of SARS-CoV-2 and SARS1 at the same region. The PRRARS motif is shown in red sticks. (*C*) SARS-CoV-2 S trimer composed of S1 subunits only. The protomers are colored orange, red, and gray, and displayed in van der Waals format. The hydrophobic, hydrophilic, acidic, and basic residues in the protruding motifs E661 to R685 are colored white, green, red, and blue, respectively. (*D*) Sequence similarity between the close neighborhood of the PRRA insert, neurotoxin motifs reported earlier (20), and HIV-1 gp120 superantigenic motif (63) in the last row.

We note that the TCRVβ-binding epitope on SARS-CoV-2 S is centered around a polybasic sequence motif, P_681_RRA_684_ (PRRA hereafter), and includes the sequential and spatial neighbors of this motif. Comparison to other β-CoV S sequences shows (19) that SARS-CoV-2 S is distinguished by the existence of this four-residue insertion, PRRA, preceding the S1/S2 cleavage site (R685−S686 peptide bond) (Fig. 2*A*). Structural comparison of the S glycoproteins between SARS1 and SARS-CoV-2 further shows their close structural similarity in general [except for their RBDs engaged in specific interfacial interactions (17)], but the two S glycoproteins significantly differ near the PRRARS motif unique to SARS-CoV-2, which is exposed to the exterior (Fig. 2*B*). Notably, the exposure of this motif and its close sequential neighbors is further accentuated in the S1 trimer (Fig. 2*C*) shed after cleavage by the human proteases (TMPRSS2 or furin) to enable the activation of the fusion trimer of S2 subunits.

### Further Examination of the Motif near PRRA Reveals Close Structural Similarity to the SEB Superantigen as well as Sequence Similarities to Neurotoxins and a Viral SAg.

The insertion PRRA together with seven sequentially preceding residues and succeeding R685 (conserved among β-CoVs) form a motif, Y_674_QTQTNSPRRAR_685_, homologous to those of neurotoxins from *Ophiophagus* (cobra) and *Bungarus genera*, as well as the neurotoxin-like regions from three RABV strains (20) (Fig. 2*D*). We further noticed that the same segment bears close similarity to the HIV-1 glycoprotein gp120 SAg motif F164 to V174. This close sequence similarity to both bacterial and viral SAgs, in support of the potential superantigenic character of the stretch Y674 to R685 of SARS-CoV-2 S, directed us to further analyze its local sequence and structure.

**Fig. 3.**
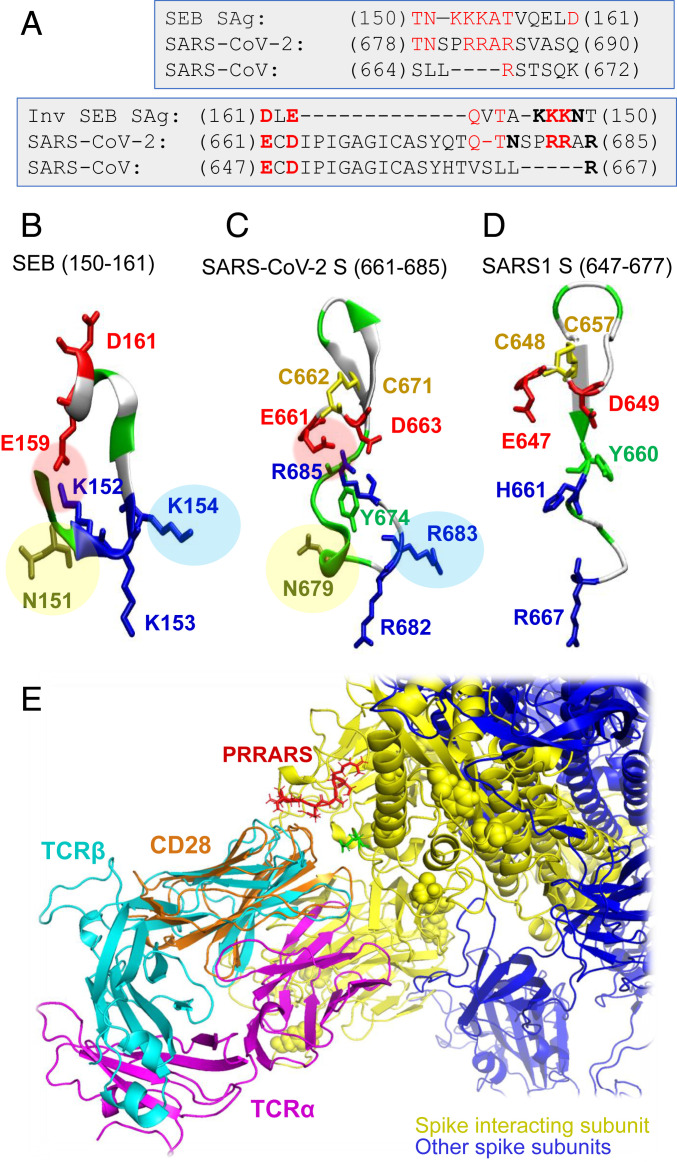
The “PRRA” insert in SARS-CoV-2 spike exhibits sequence and structure properties similar to those of the bacterial superantigen SEB. (*A*) Alignment of SEB superantigenic sequence (21) against the homologous sequence of SARS-CoV-2 spike near the PRRA insert and corresponding SARS1 S segment. Alignments are displayed for both forward (*Top*) and reverse (*Bottom*) ordering of the SEB sequence. Note the similarities between the former two, while the third (SARS1 S) shows similarities to SARS-CoV-2, but not to SEB, sequence. Charged amino acids and a critical asparagine that are structurally conserved between SARS-CoV-2 S and SEB, as illustrated in *B* and *C*, are written in boldface in the *Lower* alignment. (*B*) Structure of the superantigenic peptide (T150-D161) observed in the crystal structure of SEB (64) (PDB ID code 3SEB). (*C*) Structural model for SARS-CoV-2 S palindromic motif E661-R685. (*D*) Homologous region in SARS1 S exhibits totally distinctive structural features. Three features (highlighted by pink, blue, and yellow circles) are absent in SARS1 S. The motifs in *B* and *C* are polybasic (three lysines and three arginines in the respective cases), whereas SARS1 S counterpart has one basic residue (R667) only; the former two possess a scaffolding ASN, absent in SARS1. (*E*) Structural alignment of CD28, the receptor binding SEB, onto TCRVβ domain, in support of the adaptability of the putative SAg site to accommodate spike−TCRVβ or SEB−CD28 interactions.

Our analysis led to an interesting sequence similarity between the fragment T678 to Q690 of SARS-CoV-2 S and the SEB superantigenic peptide T_150_NKKKATVQELD_161_ (Fig. 3*A*). This dodecapeptide sequence shows strong conservation among a broad range of staphylococcal and streptococcal SAgs (21, 22). We note that the sequentially aligned segment of SARS1 (S664 to K672) bears minimal similarity to the SEB SAg (Fig. 3 *A*, left). What is even more interesting is that SARS-Cov-2 motif showed a palindromic behavior with respect to this superantigenic SEB sequence, in the sense that a broader stretch, from E661 to R685, could be aligned to the SAg peptide in the reverse direction as well (Fig. 3*A*, right). This brings to our attention the versatility and high propensity of the SARS-CoV-2 S TCRVβ-binding site residues to potentially elicit an SAg-like response.

Significantly, the structure of the SARS-CoV-2 S SAg-like segment and that of SEB peptide also exhibit a remarkable similarity (Fig. 3 *B* and *C*): A salt bridge (E159−K152 in SEB and E661−R685 in SARS-CoV-2 S) stabilizes both structural motifs; the relative orientations of three positively charged residues (K152, K153, and K154 in SEB and R682, R683, and R685 in SARS-CoV-2 S) are maintained; and an asparagine (N151 in SEB, N679 in SARS-CoV-2) completes this motif. All three features are absent in SARS1 S (Fig. 3*D*). A β-hairpin that apparently serves as a scaffold is conserved in all three spikes, and we observe a pair of cysteines that may potentially form a disulfide bond in SARS-Cov-2 and SARS1 spikes (C662−C671 and C648−C657, respectively).

This analysis overall indicates that the segment T_678_NSPRRAR_685_ may potentially form a putatively superantigenic core, consistently aligned against various bacterial or viral SAgs (Figs. 2*C* and 3 *A*–*C*) with or without the participation of the adjoining amino acids. However, combined broader sequence and structure analysis in Fig. 3 *A* (*Right*) and *B* and *C*, reveals an even more compelling feature: This putative SAg core is structurally consolidated by spatial proximity to a conserved acidic segment, E_661_CD_663_, which forms a highly stable salt bridge with the polybasic segment PRRAR of SARS-CoV-2 S, much in the same way as the salt bridge observed in SEB (but not in SARS1 S), complemented by an asparagine shared between SARS-CoV-2 S and SEB (but not SARS1 S), and the SAg character may be conferred by this type of structural scaffolding.

We note that the SEB superantigen peptide Y_150_NKKKATVQELD_161_ has been reported to bind CD28 (21), a TCR that provides costimulatory signals required for T cell activation and survival. CD28 and TCRV domains share the same (immunoglobulin, Ig) fold (Fig. 3*E*), and the binding mechanism shown in Fig. 1*B* could adapt, with minor rearrangements, to interactions with other Ig-fold molecules including neutralizing antibodies. Because of the homologous superantigenic segment of SEB binding CD28, we also tested the potential binding of SARS-CoV-2 spike residues E661 to R685 onto CD28. Our simulations indicated that the same segment can equally bind to CD28, further supporting the strong propensity of the fragment to stimulate T cell activation.

### An ICAM-1−like Motif Shared between SARS1 and SARS-CoV-2 Spikes Interacts with TCRVα to Further Stabilize the S-TCR Complex.

**Fig. 4.**
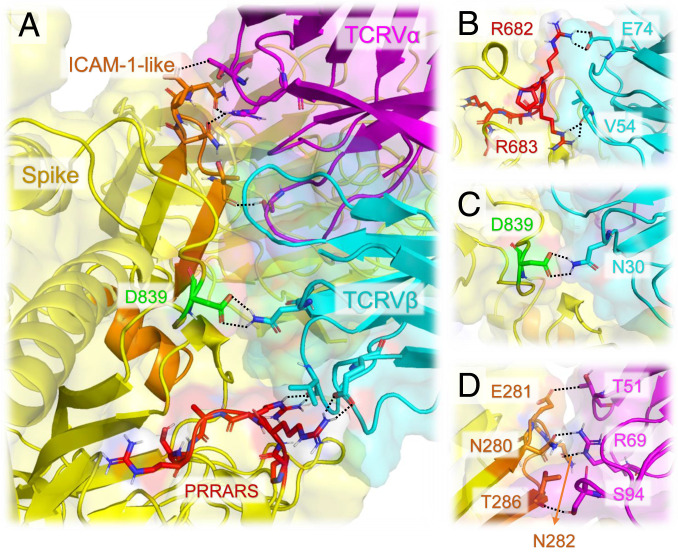
The interfacial interactions between SARS-CoV-2 spike and αβTCR are further stabilized by the association of an ICAM-1−like motif with TCRVα domain. (*A*) Interface between SARS-CoV-2 spike and TCR variable domains. Spike is shown in yellow; TCR Vα and Vβ are in magenta and cyan, respectively. The PRRARS insert is highlighted in red; the mutation site D839 identified in recent study (26) is in green; SARS-CoV-2 counterpart of ICAM-1 (CD54)-like motif identified for SARS1 spike (23) is in orange. Residues involved in close interfacial contacts are shown in sticks, with nitrogen and oxygen atoms colored blue and red, respectively. Interactions between atom pairs separated by less than 2.5 Å are indicated by black dashed lines. (*B*) A close-up view of the interactions between the PRRARS insert/motif and TCR Vβ. (*C*) Same for the D839 mutation site. (*D*) Interactions between selected residues on ICAM-1−like motif (labeled, orange) and TCRVα CDRs.

The existence of potential superantigenic, toxic, or intercellular adhesion molecule (ICAM)-like fragments in SARS1 was thoroughly examined by Li et al. (23) following the 2003 pandemic. This led to the identification of the nine sequence stretches including three *Botulinum* neurotoxin type D or G precursors and two motifs that are highly similar to ICAM-1. Comparative analysis with SARS-CoV-2 S sequence revealed that seven of these motifs are conserved (with >68% sequence identity) between SARS1 and SARS-CoV-2 spikes (*SI Appendix*, Fig. S3). Among them, the ICAM-1 (CD54)-like motif Y_279_NENGTITDAVDCALDPLSETKC_301_ also participates in the association between the SARS-CoV-2 S and αβTCR as shown in Fig. 4.

ICAM-1 involvement is critical to mediating immune and inflammatory responses. The observed interaction of the ICAM-1−like motif of SARS-CoV-2 S with TCRVα, in tandem with the interaction of the above discussed putative SAg motif (around the insert PRRA) with TCRVβ, is likely to further strengthen the association of the virus with the T cell and the ensuing activation. Precisely, T286 (S) makes close contacts with S94 (CDR3); E281 (S) forms a hydrogen bond with T51 (CDR2); and N280 and N282 (S) closely associate with R69 (Fig. 4*D*).

### A Neurotoxin-like Fragment at the RBD May also Bind αβTCR, Thus Further Enhancing the Immune Response.

**Fig. 5.**
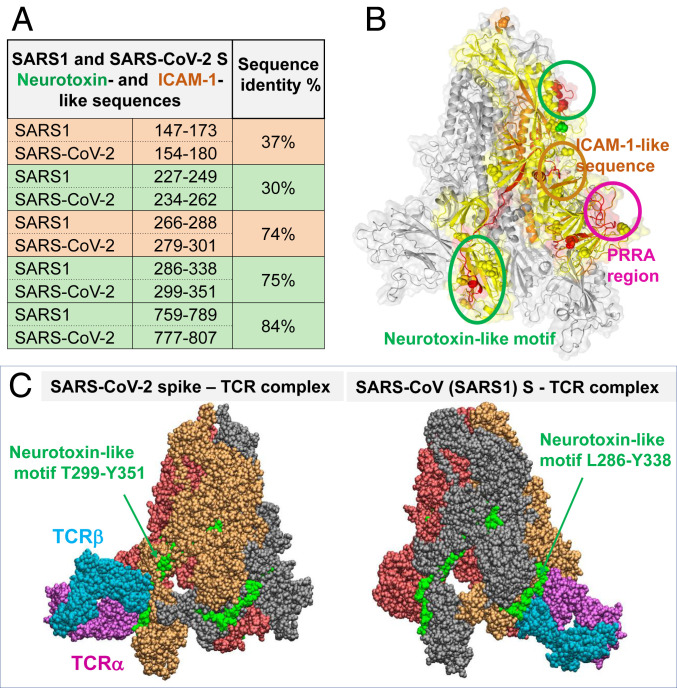
Neurotoxin-like sequences in SARS-CoV-2 S RBD and their ability to bind TCRs. (*A*) Comparison of bioactive, neurotoxin-like (green) and ICAM-1 like (orange) segments identified for SARS1 and their SARS-CoV-2 spikes. (*B*) Loci of two neurotoxin-like regions (enclosed in green circles) and one ICAM-1 region (orange circle; see Fig. 4) conserved between the two CoVs, shown on one monomer (highlighted in yellow) of SARS-CoV-2 S. (*C*) Binding poses of TCR on SARS-CoV-2 (*Left*) and SARS1 (*Right*) S proteins, making contacts with the indicated conserved neurotoxin motif.

Further examination of the SARS-CoV-2 S segments sequentially homologous to the neurotoxin-like sequences identified (23) for SARS1 S (rows colored green, Fig. 5*A*) pointed to two motifs conserved between the two CoVs: SARS-CoV-2 S1 residues 299 to 351 partially overlapping with the RBD and the S2 residues 777 to 807. Our simulations in search of possible binding of TCRs to these neurotoxin-like motifs showed the high propensity of the motif T299 to Y351 (second highest affinity site after the PRRA region) and its counterpart in SARS1 (first highest-affinity site) to bind TCRs. Fig. 5*C* illustrates these complexes.

A recent study (24) detected significant T cell reactivity against 66 epitopes on the SARS-CoV-2 S glycoprotein in people who have not been exposed to the virus, inviting attention to possible memory response acquired upon exposure to human CoVs (HCoVs) such as common cold HCoV-OC43, HCoV-HKU1, HCoV-NL63, and HCoV-229E, which share sequence homology with SARS-CoV-2 genome. A total of 142 such cross-reactive epitopes were identified upon screening 474 peptides in the SARS-CoV-2 proteome (24).

We examined whether the neurotoxin-like regions identified here were among these cross-reactive epitopes. Notably, of the top-ranking four epitopes (ranked by T cell reactivity measured by spot-forming cells [SFC]/10^6^ PBMCs), two (peptides 321 to 335 and 316 to 330) belong to the neurotoxin-like fragment T299 to Y351, and one (peptide 236 to 250) to the fragment 234 to 262. In fact, the former was completely spanned by eight partially overlapping cross-reactive epitopes as illustrated in *SI Appendix*, Fig. S4**,** pointing to the distinctive ability of this region to trigger CD4^+^ T cell response. These observations provide strong support to the predicted high affinity of this motif to bind TCR (Fig. 5*C*).

Overall, this neurotoxin-like sequence T299 to Y351 deserves attention as a possible source of central nervous system (CNS) disorders in COVID-19 patients.

Among the 66 epitopes, we note 661 to 675, which lies within the SAg-like region E661-R685 (Fig. 3), albeit at low reactivity and frequency. The absence of the insert PRRA among the cross-reactive epitopes is not surprising given that this insert is unique to SARS-CoV-2 S among all SARS-related βCo-Vs, and cross-reactivity increases with sequence similarity to antigens/peptides to which the donors have been already exposed to. The sequence identity between SARS-CoV-2 and SARS1 is 40% in the portion 671 to 685 of the SAg-like region, and the percentage of cross-reactive peptides having 33 to 40% sequence identity is reported to be 1% (24). On the other hand, it is interesting to note that, in a recent study (25) on epitopes that show strong T cell reactivity in convalescent patients who experienced severe COVID-19, an epitope (680 to 688) overlapping with the PRRA-containing part of SAg region is predicted to be one of the highest-affinity epitopes binding to HLA.

### A Rare Mutation, D839Y/E, Recently Observed in a SARS2 Strain from Europe May Contribute to Stabilizing the Interaction with TCR.

Interestingly, the SARS-CoV-2 S binding region harbors three residues that have been recently reported to have mutated in new strains from Europe and the United States (26, 27): D614G, A831V, and D839Y/N/E. The former two may potentially interact with MHCII, based on a ternary model we generated for SARS-CoV-2 S, MHCII, and TCR (*SI Appendix*, Fig. S5), while the latter **(**D839) is close to TCRVβ and strongly interacts with N30 (Fig. 4 *A* and *C* and *SI Appendix*, Fig. S6). The substitution of D839 by tyrosine strengthens the interactions between the spike and TCRVβ. The interfacial interactions in the D839Y mutant are stabilized by a hydrogen bond between Y839 and D32, an aromatic (polar-π) interaction between Y839 and N30, and possible electrostatic interactions with K73 and S97. The change in binding affinity between the spike and TCR upon mutating D839 to tyrosine is ΔΔG_D→Y_ = −0.9 ± 0.7 kcal/mol, indicating an approximate fourfold increase in binding affinity upon substituting the aspartic acid by a tyrosine at this position. The same qualitative effect is valid, but to a weaker extent, in the mutations to asparagine or glutamic acid. See *SI Appendix*, Table S1 for details on the method and results.

### TCR Repertoire Analysis Shows TCRVβ Skewing and Junctional Diversity Indicating SAg Effect in Patients with Severe and Hyperinflammatory COVID-19.

**Fig. 6.**
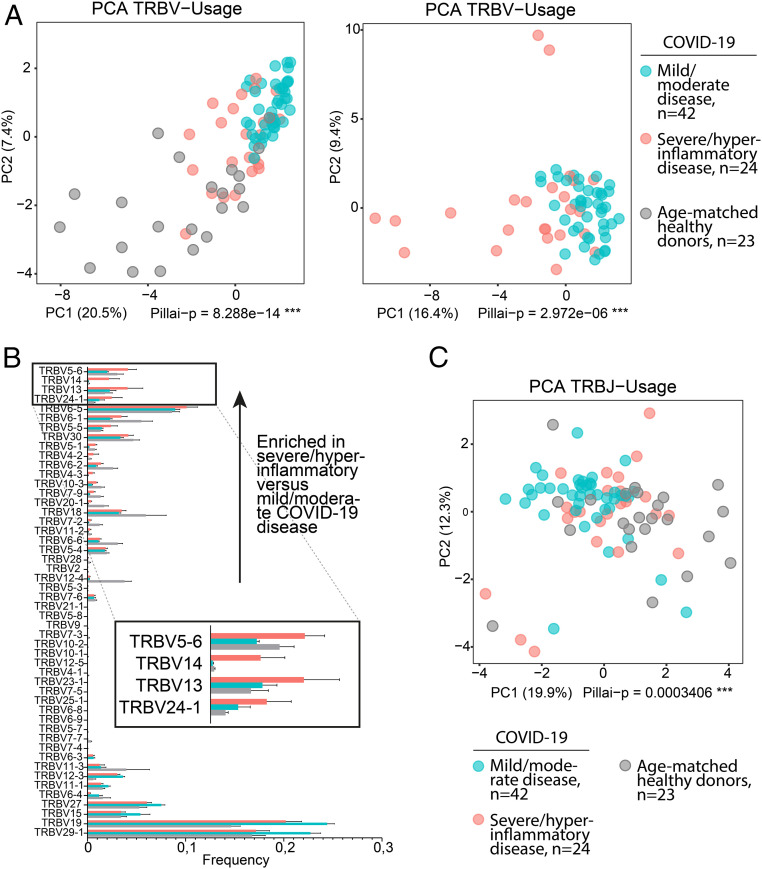
Skewing of TRBV usage in severe/hyperinflammatory COVID-19 patients; 24 repertoires of severe/hyperinflammatory COVID-19 cases versus 42 repertoires of mild/moderate COVID-19 cases were analyzed with and without 23 repertoires of age-matched healthy donors (age-matched to severe/hyperinflammatory COVID-19 group). (*A*) PCA of TRBV usage. Principal components 1 and 2 are shown; percentage of axis contributions are given in parentheses. Statistical analysis was performed using multivariate analysis of variance (MANOVA) Pillai–Bartlett test. (*B*) TRBV usage. The fraction of individual TRBV genes per repertoire is shown as mean ± SEM. TRBV genes are sorted to enriched fractions in severe/hyperinflammatory versus mild/moderate COVID-19 disease in ascending order from bottom to top. The top TRBVs enriched in severe/hyperinflammatory COVID-19 patients (TRBV5-6, TRBV14, TRBV13, and TRBV24-1) are enlarged in *Inset*. (*C*) PCA of TRBJ usage as described in *A*. See also *SI Appendix*, Fig. S7.

SAg binding to specific TCR Vβ chains results in Vβ skewing, such that T cells with specific Vβ chains and diverse antigen specificity dominate the TCR repertoire (11, 13). If the motif we identified in SARS-CoV-2 S acts as an SAg, we reasoned that patients with mild/moderate COVID-19 disease courses and recovery without hyperinflammation would show adaptive immune responses mediated by T cells recognizing SARS-CoV-2 epitopes in a CDR3-mediated fashion, whereas patients with severe/hyperinflammatory COVID-19 would show immune responses consistent with at least partial SAg recognition. We analyzed, to this aim, next generation sequencing (NGS) immunosequencing data from 38 patients (42 samples) with mild/moderate COVID-19 and 8 patients (24 samples) with severe, hyperinflammatory COVID-19, which were part of a previously studied cohort (28). Principal component analysis (PCA) of the TCR β-chain variable gene (TRBV) repertoires corresponding to the two groups revealed that patients with mild/moderate COVID-19 course clustered apart from those with severe/hyperinflammatory COVID-19 (Fig. 6*A*).

Differential gene usage analysis showed that several TRBV genes were overrepresented in the severe/hyperinflammatory COVID-19 patient group (Fig. 6*B*). In contrast, PCA of J gene distribution showed much less skewing, suggesting a selective pressure was preferentially exerted on the V gene distribution (Fig. 6*C*). To further investigate J gene diversity specifically for the V genes overrepresented in the severe/hyperinflammatory COVID-19 cases, we extracted all J genes rearranged with TRBV5-6, TRBV13, TRBV14, and TRBV24-1 from the repertoires of severe/hyperinflammatory COVID-19 patients and compared this to J genes extracted from the age-matched healthy donors. This analysis showed very diverse TRBJ gene distribution, implying CDR3 diversity in the respective expanded rearrangements (*SI Appendix*, Fig. S7).

Together, our results suggest that patients with severe and hyperinflammatory COVID-19 show expansion of TCRs using distinct V genes, along with J gene/CDR3 diversity in these rearrangements, compatible with an SAg selection process.

### TCRs Corresponding to TRBV Genes Activated in Severe COVID-19 Patients Can Bind to the SAg-like Region of SARS-CoV-2 S.

Finally, we turned our attention to structurally resolved TCRs that contained Vβ chains encoded by the genes TRBV5-6, TRBV13, TRBV14, and TRBV24-1 enriched in severe/hyperinflammatory COVID-19 patients. We tested whether these TCRs could bind the SAg-like region E661 to R685 of the SARS-CoV-2 S similarly to the TCR in Fig. 1. Our Protein Data Bank (PDB) search yielded αβTCR structures corresponding to TRBV5-6, TRBV14, and TRBV24-1, that is, TCRs whose Vβ chains were 95 to 100% identical to the protein product of these three genes. As shown in *SI Appendix*, Fig. S8**,** all three were verified to bind the SAg-like site with high affinity, and to make interfacial interactions closely resembling those illustrated in Fig. 1. Our models and simulations also indicated the possibility of energetically favorable ternary complex formation between these TCRs, MHCII, and spike. Overall, these simulations showed that these TCRs enriched in severe/inflammatory COVID-19 patients could bind the SARS-CoV-2 S at its SAg-like region and form ternary complexes with MHCII.

## Conclusion

An understanding of the immunopathology leading to severe manifestations of COVID-19, in both adults and children, is of critical importance for effective management and treatment of the disease. MIS-C shows remarkable similarity to pediatric TSS (5–9). Using in silico modeling and analysis, we found that SARS-CoV-2 encodes a superantigen motif near its S1/S2 cleavage site. This region is highly similar in sequence and structure to the SEB SAg motif that interacts with both the TCR and CD28 (21) and mediates TSS. SEB enables large-scale T cell activation and proliferation (12), resulting in massive production of proinflammatory cytokines including IFNγ, TNFα, and IL-2 from T cells, as well as IL-1 and TNFα from antigen-presenting cells (12). This cytokine storm leads to multiorgan tissue damage like what is now observed in MIS-C. Our results suggest that the hyperinflammatory syndrome originates from superantigenic activity by SARS-CoV-2 S glycoprotein. Furthermore, these findings also raise the possibility that the hyperinflammation observed in severe cases of COVID-19 in adults may also be driven by the SAg-like activity of the S protein. Indeed, SAgs induce an inflammatory cytokine signature similar to that which predicts severity and death in COVID-19, including IL-6, TNFα, IL-8, and IL-1β (12, 29). Moreover, our analysis of the T cell immune response in COVID-19 patients shows that those with more severe and hyperinflammatory clinical courses exhibit TCRVβ skewing consistent with SAg activity.

Interestingly, a thorough study of SARS1 immunogenicity, conducted with a cohort of 128 individuals who have recovered from SARS1 (30), showed that the SARS1 spike 18-mer D649-L666 (DIPIGAGICASYHTVSLL) is one of the peptides most frequently recognized by T cells, among the screened 1,843 peptides that span the whole SARS1 CoV proteome [table 3 in Li et al. (30)]. This segment coincides with the SARS1 S region E647 to R667 that is sequentially (and structurally) homologous to our SARS-CoV-2 spike SAg-like motif E661 to R685 (Fig. 3*A*, bottom alignment). This provides a very strong support for the T cell stimulatory ability of our SAg motif, given that it shares 12/18 amino acids with that SARS1 18-mer. And the remaining amino acids (including the insert PRRA, not present in SARS1 S) would endow even stronger superantigenic properties by virtue of their close similarity to the aligned SEB fragment.

Our findings raise the exciting possibility that immunomodulatory therapeutic options used for TSS may also be effective for MIS-C, including intravenous immune globulin (IVIG) and steroids. Indeed, initial published and unpublished reports suggest that MIS-C patients respond well to IVIG with or without steroids (5–7). IVIG contains antibodies that neutralize SEB (31). Given structural similarities between SEB and the SARS-CoV-2 S protein SAg motif, there is potential for cross-reactivity of these immunoglobins, which may, in part, explain the response of MIS-C cases to IVIG. Other Food and Drug Administration-approved antiinflammatory drugs tested in models of SEB TSS may also be effective, including CTLA4-Ig which can inhibit CD28 costimulation (32), and the mammalian target of rapamycin (mTOR) inhibitor rapamycin (33), which is already in use for COVID-19. In addition, humanized monoclonal anti-SEB Abs (34, 35) could be of potential therapeutic benefit in MIS-C patients. Notably, it has been shown, in the mouse model of TSS, that lethal SEB superantigen challenge can be prevented by short peptide mimetics of its superantigen motif (21). It would be interesting to examine whether short peptide mimetics of the SARS-CoV-2 spike superantigen region might be employed to prevent/attenuate inflammatory cytokine gene induction and toxic shock in MIS-C patients.

At present, the majority of COVID-19 antibody therapies under investigation are designed to target the RBD, and some the N-terminal domain (NTD), of SARS-CoV-2 spike (36–44). Our simulations also indicate that the RBD might potentially interact with TCRs. However, our study suggests that the “stem” of the spike, or its SAg-like motif, might also serve as a target. Compared with RBDs, relatively fewer mutations are found in the SAg region of SARS-CoV-2; notably, the PRRA insert is unique to SARS-CoV-2 and retained among all of its isolates sequenced to date (26, 27). It is also important to note that most of the cryo-EM structures reported for SARS-CoV-2 S protein have GSAS or GSGS substitution at R_682_RAR_685_, following the original work by Wrapp et al. (17). This same segment is either mutated (42–45) or removed (38–41) to enable protein expression and/or resolution, in cryo-EM studies of antibody-, nanobody-, or Fab-bound spike. The “mutant spike” is thus devoid of the unique character that would be otherwise endowed by the polybasic insert P_681_RRA and adjacent cleavage site R_685_S, and the high reactivity of R_682_RAR_685_ may have eluded these studies. It might be constructive to design antibodies or drugs targeting this SAg region, to not only modulate the SAg-induced inflammatory cytokine gene induction (12) but also block the cleavage essential to enabling viral entry (1, 19). Alternatively, combination therapies that target both the SAg-like region and the RBD can prove useful.

Fortunately, severe respiratory manifestations of COVID-19 in children as well as development of MIS-C are rare. This may be due to trained immunity (2). T and B cells play an important role in the antiviral response. CD4+ and CD8+ T cells from convalescent COVID-19 patients can recognize a range of SARS-Cov-2 epitopes, and the S protein represents a major target. Interestingly, T cells from unexposed individuals also respond to S protein epitopes from SARS-CoV-2, which supports the hypothesis of cross-viral immunity from other coronavirus strains (24, 46). However, why only a fraction of infected children develop MIS-C is unclear. We show that the mutation D839Y found in a European strain of SARS-CoV-2 enhances the binding affinity of the SAg motif to the TCR. This could (at least partly) explain the geographical skewing of MIS-C to areas where the European strain is endemic. It is also possible that a poor initial antibody response to the virus fails to neutralize the SAg, as recently shown in MIS-C patients (47), leading to immune enhancement following reexposure. Certain HLA types are more permissive of binding SAg, and, indeed, HLA has been shown to play a role in COVID-19 susceptibility (48). Of the nine cases initially reported in the United Kingdom, six were of Afro-Caribbean descent, which also suggests a potential genetic component to susceptibility (5). In addition, ∼80% of individuals over age 12 y harbor anti-SEB antibodies (49, 50), which may provide protection against the SAg effects of SARS-CoV-2 S protein. The prevalence of preexisting anti-SEB antibodies could also contribute to the age distribution of severe COVID-19 cases in adults, as protective SEB titers fall in older adults after age 70 y.

It is interesting to note that approximately a third or fewer of MIS-C patients tested positive for SARS-CoV-2, but the majority (but not all) had serologic evidence of infection or a history of exposure to COVID-19 (5−7). This may suggest that the SARS-CoV-2 SAg causes a delayed hyperinflammation response in certain children. SAgs have been implicated in autoimmunity by triggering self-reactive T cells (11). Antibody-mediated enhancement upon reexposure to the virus may also contribute to uncontrolled infection and inflammation (51). It is also possible that, despite a negative nasopharyngeal PCR test, the virus may still be present in the GI tract (52). MIS-C patients demonstrate unusually severe GI symptoms, abdominal pain, vomiting, and diarrhea, in addition to severe myocardial dysfunction and cardiac shock (5–7) and such severe GI symptoms are also frequently associated with the response to SAgs (9). In the case of SEB, cleavage and release of a specific fragment is responsible for induction of GI symptoms. Whether the SARS-CoV-2 SAg-like structure that we discovered could be similarly cleaved and would underlie the GI symptoms observed in MIS-C patients remains to be determined.

We also observed that a neurotoxin-like segment (T299 to Y351) partially overlapping with the RBD exhibited a high affinity to bind TCRs. Notably, this region was recently observed to elicit strong and frequent T cell reactivity mediated by CD4+ T cells in donors who have not been exposed to SARS-CoV-2 (24). This invites attention to its ability to potentially trigger neurotoxic immune response in individuals who have not been exposed to CoVs that contain sequentially homologous peptides. Moving forward, it will be important to establish the significance of this region in the neurological disorders that are also frequently reported in children with MIS-C as well as adults.

In summary, we made five major observations: 1) PRRAR and sequential neighbors interact with TCRVβ CDRs, and this association closely resembles that of SEB SAg with TCRVβ; 2) nearby D839 participates in this interaction, and its mutation to tyrosine could strengthen the association with TCRVβ; 3) a sequence motif (N280 to T286) typical of ICAM-1 interacts with the TCRVα, further stabilizing the association between the spike and host cell TCR; 4) a neurotoxin-like motif (T299 toY351) shows a high tendency to bind TCRs and potentially trigger neurotoxic responses, and this latter effect may be attenuated if the SARS-CoV-2−infected individual has been exposed HCoVs that contain homologous segments, as suggested (24) by a recent study; and 5) adult patients with severe/hyperinflammatory COVID-19 exhibit a skewed TCR Vβ repertoire distinguishing them from patients with mild/moderate COVID-19. Overall, our results from both computational modeling and NGS immunosequencing of TCRBs analysis of human samples indicate that strategies used for the treatment of SEB-mediated TSS or approaches to block the interaction of the S protein with TCRs may help reduce hyperinflammatory manifestations or (neuro)toxic effects of COVID-19 in both adults and children.

## Materials and Methods

SARS-CoV-2 (P0DTC2) and SARS-CoV (CVHSA_P59594) spike models were generated using SWISS-MODEL (53), based on the resolved spike glycoprotein structures of SARS-CoV-2 (17) (PDB ID code 6VSB) and SARS-CoV (54) (PDB ID code 6ACD).The missing loops in the crystal structures were built using libraries of backbone fragments (55) or by constraint space de novo reconstruction of these backbone segments (56). Two mutants associated with European COVID-19 patients (26) were constructed using CHARMM-GUI (57): one is the main strain mutant D614G, and the other contains four mutations including Q239K, A831V, D614G, and D839Y. These two SARS-CoV-2 spike mutants together with the SARS-CoV-2 (P0DTC2) originally taken from Wuhan were used to investigate the binding to αβTCR, and MHCII (PDB ID code 2XN9) (16) using ClusPro (18) and PRODIGY (58). See details in *SI Appendix*. The NGS immunosequencing data were analyzed using the methods described earlier (59, 60), summarized in *SI Appendix*.

## Supplementary Material

Supplementary File

## Data Availability

All study data are included in the article and *SI Appendix*.
