# Supplementary material for: Superantigenic character of an insert unique to SARS-CoV-2 spike supported by skewed TCR repertoire in patients with hyperinflammation
Source: Proc Natl Acad Sci U S A. 2020 Sep 28;117(41):25254–62. doi: 10.1073/pnas.2010722117 (PMC7568239; doi:10.1073/pnas.2010722117)
Supplement: Supplementary File [file pnas.2010722117.sapp.pdf]

Superantigenic character of an insert unique to SARS-CoV-2 spike supported by skewed TCR repertoire in patients with hyperinflammation

Mary Hongying Cheng[1], She Zhang[1], Rebecca A. Porritt[2,3], Magali Noval Rivas[2,3], Lisa Paschold[4], Edith Willscher[4], Mascha Binder[4], Moshe Arditi[2,3] and Ivet Bahar[1]*

[1]Department of Computational and Systems Biology, School of Medicine, University of Pittsburgh, Pittsburgh, PA 15213, Departments of [2]Pediatrics, Division of Pediatric Infectious Diseases and Immunology, [3]Biomedical Sciences, Infectious and Immunologic Diseases Research Center, Cedars-Sinai Medical Center, Los Angeles, CA 90048. [4]Department of Internal Medicine IV, Oncology/Hematology, Martin-Luther-University Halle-Wittenberg, 06120 Halle (Saale), Germany

**Corresponding author*

Dr. Ivet Bahar
Distinguished Professor and John K. Vries Chair
Department of Computational & Systems Biology
School of Medicine, University of Pittsburgh
3064 Biomedical Science Tower 3
3501 Fifth Avenue, Pittsburgh, PA 15213
Voice: 4126483332 - Fax: 4126483163
bahar@pitt.edu; http://www.ccbb.pitt.edu/Faculty/bahar/

www.pnas.org/cgi/doi/10.1073/pnas.2010722117

Supplemental Information and Supplemental Figures

Generation of a binary complex between SARS-CoV-2 spike and T cell receptor (TCR)

SARS-CoV-2 spike model in the prefusion state was generated using SwissModel (1) based on the resolved cryo-EM structure (Protein Data Bank (PDB): 6VSB) (2) for the S glycoprotein where one of the receptor binding domains (RBDs) is in the up conformation and the other two in the down conformation. The structure of the T cell receptor (TCR) containing both α- and β-chains was taken from the crystal structure (PDB: 2XN9) of the ternary complex resolved for human TCR, *staphylococcal* enterotoxin H (SEH) and human major histocompatibility complex class II (MHCII) molecule (3). Using protein-protein docking software ClusPro (4), we constructed *in silico* a series of binary complexes for SARS-CoV-2 spike and TCR. We obtained 30 clusters of conformations for spike-TCR binary complexes, upon clustering the ~1000 models generated by ClusPro. The clusters were rank-ordered by cluster size, as recommended (4). We analyzed all models and found that a large fraction showed that TCR bound to spike via its constant domain. Given that the constant domain is proximal to the cell membrane and TCR employs the variable domain for binding superantigens (SAgs) and/or antigen/MHC complexes (3), we then added restraints to our docking simulations to filter out those conformers where the variable domain would bind to the spike. This led to 27 clusters (based on a set of 666 models) from ClusPro. Interestingly, in 45% of the generated models, the TCR was observed to bind to a spike epitope that contained the “PRRA” insert; and in 46% of models we observed an interaction between the TCR and one or two of the three RBDs.

Thus, we identified two hot spots for TCR binding within the SARS-CoV-2 spike: one overlapping with the “PRRA” insert and the other on the RBD surface. Representative members belonging to the top-ranking clusters are presented in **Fig. S1**. Panels **A** and **B** illustrate two cases where the TCR tightly binds to the PRRA insert region (of monomers 2 (*dark red*) and monomer 1 (*gray*), respectively); and panels **C** and **D** illustrate two cases where the TCR binds to RBDs.

Generation of a binary complex between SARS-CoV spike and TCR

SARS-CoV (SARS1) spike model in the prefusion state was generated using SwissModel (1) based on the cryo-EM structure resolved for SARS-CoV spike (PDB: 6ACD) (5) where one of the RBDs is in the up conformation, and the other two in the down conformation. Following the same approach as we did for SARS-CoV-2 spike, we constructed *in silico* a series of binary complexes for SARS-CoV spike and TCR using ClusPro (4). Using the same filtering procedure, this led to 30 clusters (based on 686 models), among which 38% showed the binding of TCR to multiple RBDs (see **Fig. S2A**) similar to the behavior observed (in **Fig. S1C-D**) for SARS-CoV-2. Differently, 48% of the models showed the binding of TCR to two S2 subunits near the C-terminal domain of the trimers (see **Fig. S2B**). No significant binding of TCR near the S1/S2 cleavage site RS_{668} of the SARS1 spike was observed. Note that the residues $S_{664}LLRS_{668}$ of SARS1 spike, which are sequentially aligned against the SARS-CoV-2 spike $T_{678}SPRRARS_{686}$ containing the “PRRA” insert (see **Fig. 3** panel **A**), lack the polybasic character of their counterpart SARS-CoV-2. The lack of TCR

binding to this region is consistent with the absence of this motif in SARS-CoV, which serves as a strong attractor in SARS-CoV-2.

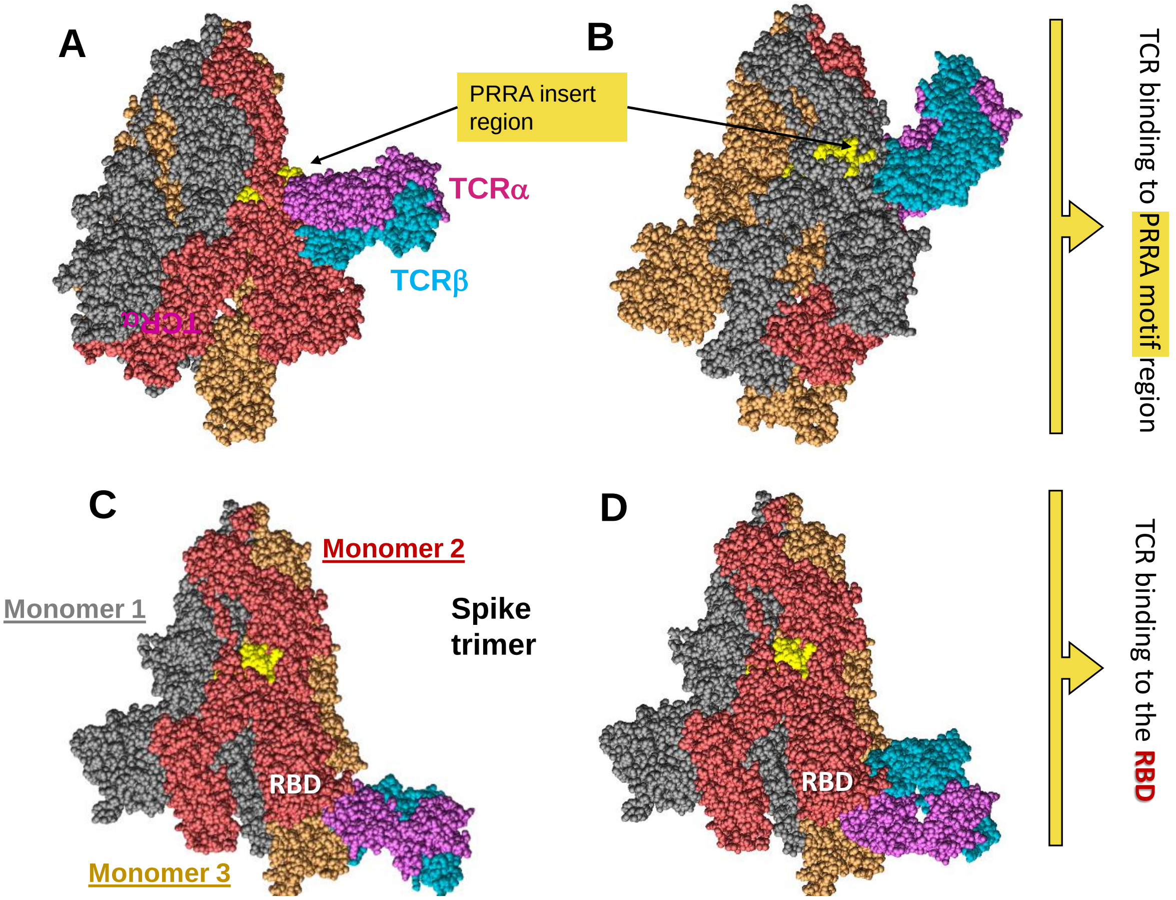

Figure S1: Top-ranking complexes between SARS-CoV-2 spike and T-cell receptor (TCR) predicted by ClusPro. (**A-B**) Binding of TCR near the "PRRA" insert region of monomer 2 (*dark red*) and monomer 1 (*gray*) in the respective panels **A** and **B**, showing that the PRRA insert and its close vicinity presents a high-affinity binding site for TCR. (**C-D**) Binding of TCR near the RBD of a subunit, indicating that the RBD is an alternative high-affinity site. The spike trimer subunits are colored *dark red*, *orange,* and *gray*. The PRRA insert region (E661 to R685) is colored *yellow*. The TCR α- and β-chains are shown in *magenta* and *cyan*, respectively. See more details on the interaction between the PRRA insert region and TCR in **Fig. 1**.

Generation of a binary complex between MERS-CoV spike and TCR

MERS-CoV spike model was generated using SwissModel (1) based on the cryo-EM structure resolved for MERS-CoV spike (6) (PDB: 5X5F) in which one of RBDs is in the up conformation. 30 clusters (based on 588 models) were predicted by ClusPro (4). 56% of models led to TCR binding to the RBDs. Two representative poses from these most populated clusters are shown in **Figs. S2 C-D**, which are comparable to those observed in SARS-CoV-2 spike (**Fig. S1C-D**) and SARS-CoV spike (**Fig. S2A**). Simulations also indicated that TCR could bind near the S1/S2 cleavage site region of MERS-CoV spike (segment D726- R751; counterpart of SARS-CoV-2 E661-R685 at the C-terminus of subunit S1). Note that at this region the PRRA insert of SARS-CoV-2 spike is replaced by MERS-CoV spike sequence PRSV. The region near PRSV shows a tendency to bind TCR but it is weaker than that of SARS-CoV-2 spike due to the lack of the critical residues (e.g. N679 and R683 in SARS-CoV-2 spike) that are involved in the interface the spike makes with the TCR. The lack of polybasic residues at this sequence motif, as well as counterparts of N679 and R683 of SARS-CoV-2 spike renders this structural region less attractive to TCRs, suggesting that MERS-CoV might not harbor a superantigen-like motif near its S1/S2 cleavage site.

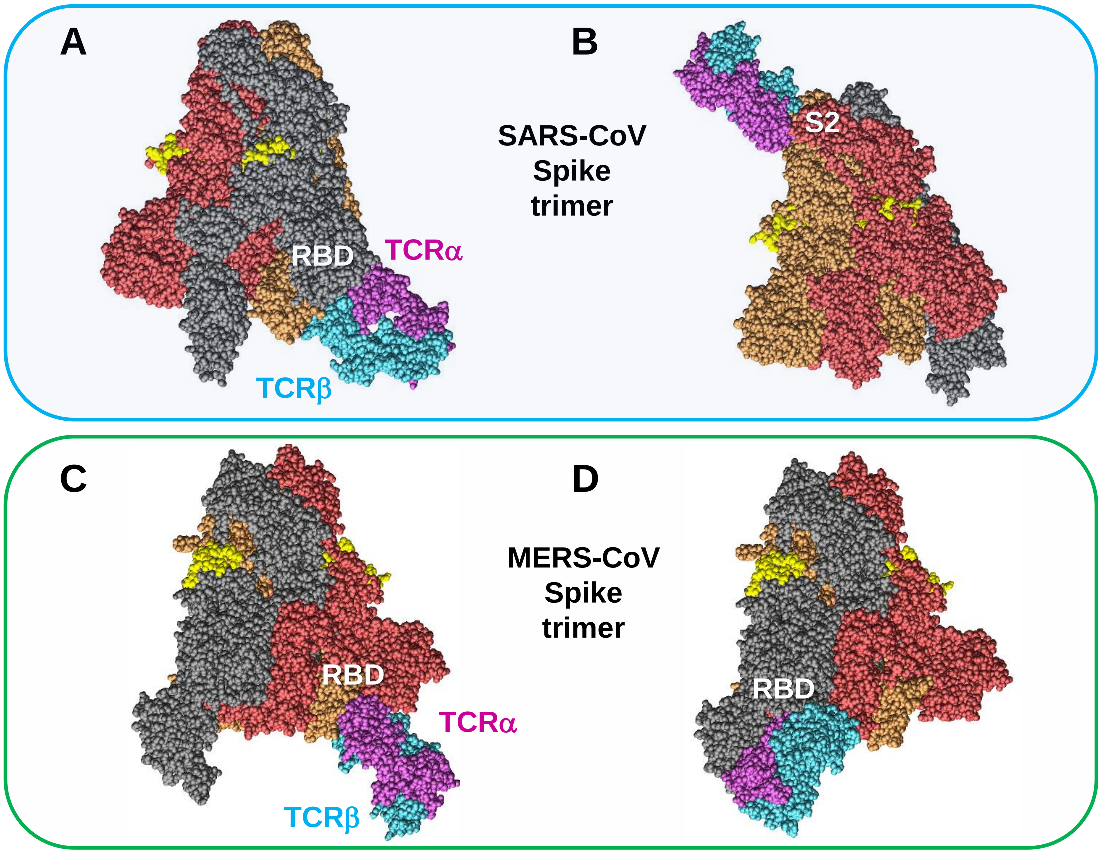

Figure S2: Complex conformers formed upon binding of TCR onto SARS-CoV spike (A-B) and MERS-CoV spike (C-D), representative of the most probable clusters predicted by docking simulations. (**A-B**) Top two TCR binding poses predicted for SARS-CoV spike: one within the RBDs and the other within S2 subunits near the C-terminal domains. (**C-D**) Top two TCR binding poses predicted for MERS-CoV: one

between the RBDs in the down conformation and the other on the RBD of the up conformation. The cleavage region in SARS-CoV (E647 to R667) and MERS-CoV (D726 to R751) are shown in *yellow*. TCR α- and β-chains are shown in *magenta* and *cyan*. Docking simulations were performed using ClusPro.

Examination of neurotoxin-like and other bioactive segments on SARS-CoV-2 spike

Fig. S3 displays nine SARS-CoV segments that have been identified to be bioactive, neurotoxin-like or ICAM-like. In each case the 2nd row is the SARS-CoV-2 segment, identified by Li et al (8) and the 1st row is its SARS-CoV-2 spike counterpart. The last row lists their percent sequence identity.

Bioactive (SAg/toxic/ICAM-like) sequences of SARS-Cov S protein (*1st row*) and corresponding homologous sequences in SARS-CoV-2 spike (*2nd row*)		Residues	Seq id
**1**	VIPFKDGIYFAATEKSNVVRGWVFGSTM	80-107	68%
	VLPFNDGVYFASTEKSNIIRGWIFGTTL	83-110	
**2**	QTHTMIFDNAFNCTFEYISDAFSLDVS	147-173	37%
	ESEFRVYSSANNCTFEYVSQPFLMDLE	154-180	
**3**	NITNFRAILT---AF-SPAQDI---WGTSA	227-249	30%
	NITRFQTLLALHRSYLTPG-DSSSGWTAGA	234-262	
**4**	YDENGTITDAVDCSQNPLAELKC	266-288	74%
	Y**NEN**GTI**T**DAVDCALDPLSETKC	279-301	
**5**	LKCSVKSFEIDKGIYQTSNFRVVPSGDVVRFPNITNLCPFGEVFNATKFPSVY	286-338	75%
	TKCTLKSFTVEKGIYQTSNFRVQPTESIVRFPNITNLCPFGEVFNATRFASVY	299-351	
**6**	GCLIGAEHVDTSYECDIPIG	634-653	90%
	GCLIGAEHVNNSYECDIPIG	648-667	
**7**	NTREVFAQVKQMYKTPTLKYFGGFNFSQILP	759-789	84%
	NTQEVFAQVKQIYKTPPIKDFGGFNFSQILP	777-807	
**8**	EAEVQIDRLITGRLQSLQTYVTQQLIRAAEIRASANLAATKMSECVLGQSKRVDFCGKGYHLMS FPQAAPHGVVFLHVTYVPS	970-1052	98%
	EAEVQIDRLITGRLQSLQTYVTQQLIRAAEIRASANLAATKMSECVLGQSKRVDFCGKGYHLMS FPQSAPHGVVFLHVTYVPA	988-1070	
**9**	LQPELDSFKEELDKYFKNHTSPDVDLGDISGINASVVNIQKEIDRLNEVAKNLNESLIDLQ	1123-1183	100%
	LQPELDSFKEELDKYFKNHTSPDVDLGDISGINASVVNIQKEIDRLNEVAKNLNESLIDLQ	1141-1201	

Figure S3: SARS-CoV-2 spike sequence fragments homologous to nine SARS-CoV spike sequence motifs related to neurotoxins and other bioactive molecules, and their location in the 3D structure. Two aligned sequences are shown for each of the 9 motifs numbered in column 1: SARS-CoV spike sequence (*top*) and SARS-CoV-2 spike sequence (*bottom).* The SARS-CoV motifs have been identified (8) by Li et al (2003) to be related to neurotoxins (row #s 3, 5 and 7; colored *green*), intercellular adhesion molecule (ICAM)-1 like protein (rows # 2 and 4; colored *orange*), *bullous pemphigoid* antigen 1-e (row # 9; *white*) and other bioactive molecules (remaining rows). We display the optimally aligned SARS-CoV-2 sequence under the SARS-CoV sequence in each of the 9 motifs, as well as the corresponding residue ranges. The last column shows the sequence identity between the two sequence fragments. The conserved ICAM-I like motif (residues 279-301 in SARS-CoV-2 spike) contains residues that interact with TCR Vα (marked in *red*). See **Fig. 5** for more information on the neurotoxin-like motif in row 5.

Note that the neurotoxin-like sequence #5, residues 299-351, contains several fragments (15-mers) that were recently shown (7) to stimulate T cell reactivity (illustrated in **Fig. S4**).

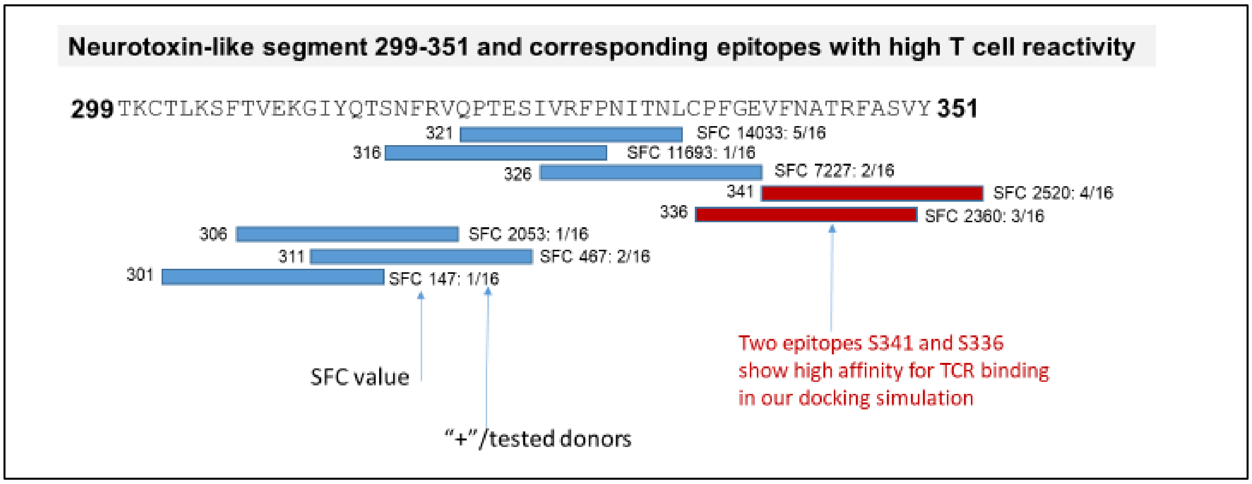

Figure S4: Position of SARS-CoV-2 S cross-reactive epitopes identified (7) in people who have not been exposed to SARS-CoV-2, which overlap with the neurotoxin-like fragment 299-355 identified here to have a strong affinity to bind TCRs. The positions of eight cross-reactive epitopes (15-mers each, with the starting amino acid shown in each case) that were recognized by CD4+ T cells are indicated by *blue* and *red bars*. In each case the corresponding reactivity strength (SFC/10^6 cells) and the number of donors (out of a total of 16) who showed this type of 'memory' response (presumably due to earlier human coronavirus infections) are written. Two of the epitopes were found in our docking simulations to bind TCRs (see **Fig 5**). Note that this is one of three neurotoxin-like regions on SARS-CoV-2 spike (highlighted in *green* in **Figs S3** and **5A**). The other two regions also contained epitopes that were cross-reactive, but this one was distinguished by its high frequency (fraction of donors) and high strength (SFC).

Generation of a ternary complex between SARS-CoV-2 spike, TCR, and MHCII

The 3-dimensional structure of the human MHCII was taken from the crystal structure of the ternary complex (3) (PDB: 2XN9) between human TCR, SEH and MHCII. First, we performed docking simulations to generate binary complexes between MHCII and SARS-CoV-2 spike. Six representative MHCII-spike binary complexes were selected to explore further docking of TCR to form a ternary complex. We analyzed all predicted ternary complex models of MHCII-Spike-TCR. Potential ternary MHCII-Spike-TCR complex models were selected following three filtering criteria: (i) TCR binds the "PRRA" insert region or the RBD; (ii) the binding region preferably includes one or more of SARS-CoV-2 spike segments that are sequentially homologous to the superantigen or toxin binding motifs predicted for SARS-CoV (**Fig. S3**); (iii) MHCII and TCR are in close proximity. These filters led to the MHCII-Spike-TCR complex model illustrated in **Fig. S5A**. Interestingly, the SARS-CoV-2 spike binding region harbors three residues that have been recently reported to have mutated in new strains from Europe and USA(9, 10) (**Fig. S5B**): D614G, A831V and D839Y/N/E). While we do not exclude the possible occurrence of other potential ternary

complexes, especially those involving the RBDs, we focused here on the complex shown in **Fig. S5**, which uniquely satisfied the three criteria.

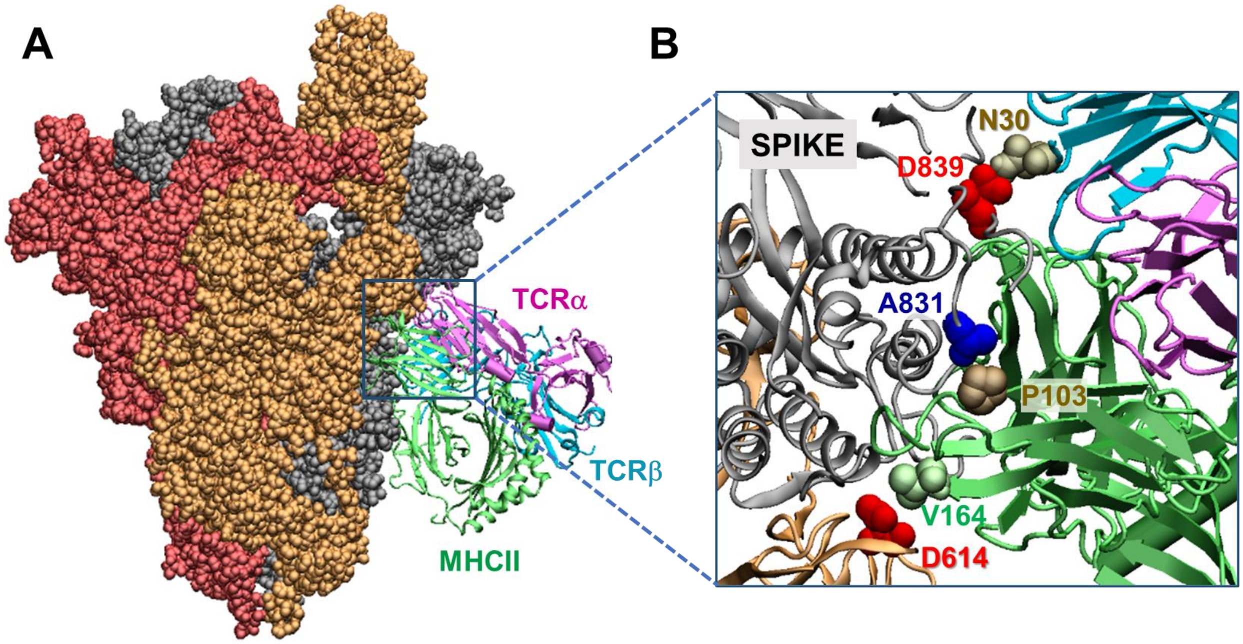

Figure S5: **Modeled ternary complex formed by SARS CoV-2 spike, MHCII and αβTCR**. (**A**) Side view of the complex. The trimeric spike subunits are shown in the same style and color as in **Fig. S1**, as well as TCRα and TCRβ. The TCR retains a similar pose as in panel **B** in **Fig. S1**, now rotated by 180˚ along the z-axis. MHCII is displayed in *green* ribbon diagram. (**B**) Top view of the interfacial contacts. Note that three spike residues reported to mutate in recent strains observed in European and western counties(9, 10) (D614G, A831V and D839Y/N/E) are within 3-5 Å from either MHCII or TCRVβ.

In silico mutagenesis of D839 of SARS-CoV-2 spike

We mutated D839 of the SARS-CoV-2 spike *in silico* to asparagine, glutamic acid and tyrosine in line with the aforementioned mutants D839Y/N/E observed in a new strain from Europe. To this aim, we used PyMOL mutagenesis tool(11) and evaluated the change in local conformation and energetics in the complex formed with TCR. The most probable rotamers were selected and energetically minimized in the presence of the bound TCR (conformation shown in **Fig. 1**) using OpenMM(12). The resulting conformations are shown in **Fig. S6**. These were further subjected to short (1 ns) molecular dynamics (MD) simulations for equilibration and energy minimization under the AMBER14 ff14SB forcefield(13). Five independent runs were carried out for each mutant (Y, N, or E, at the position 839) as well as the wild type (D839) spike, to assess the statistical significance of the results for each case. Binding affinities (ΔG) were evaluated for the final conformations of (i) the full complex (with the intact spike and entire TCR as interactors) or

(ii) a single spike subunit and TCRVβ, at 37 °C using PRODIGY server(14, 15). The results are presented in **Table S1**.

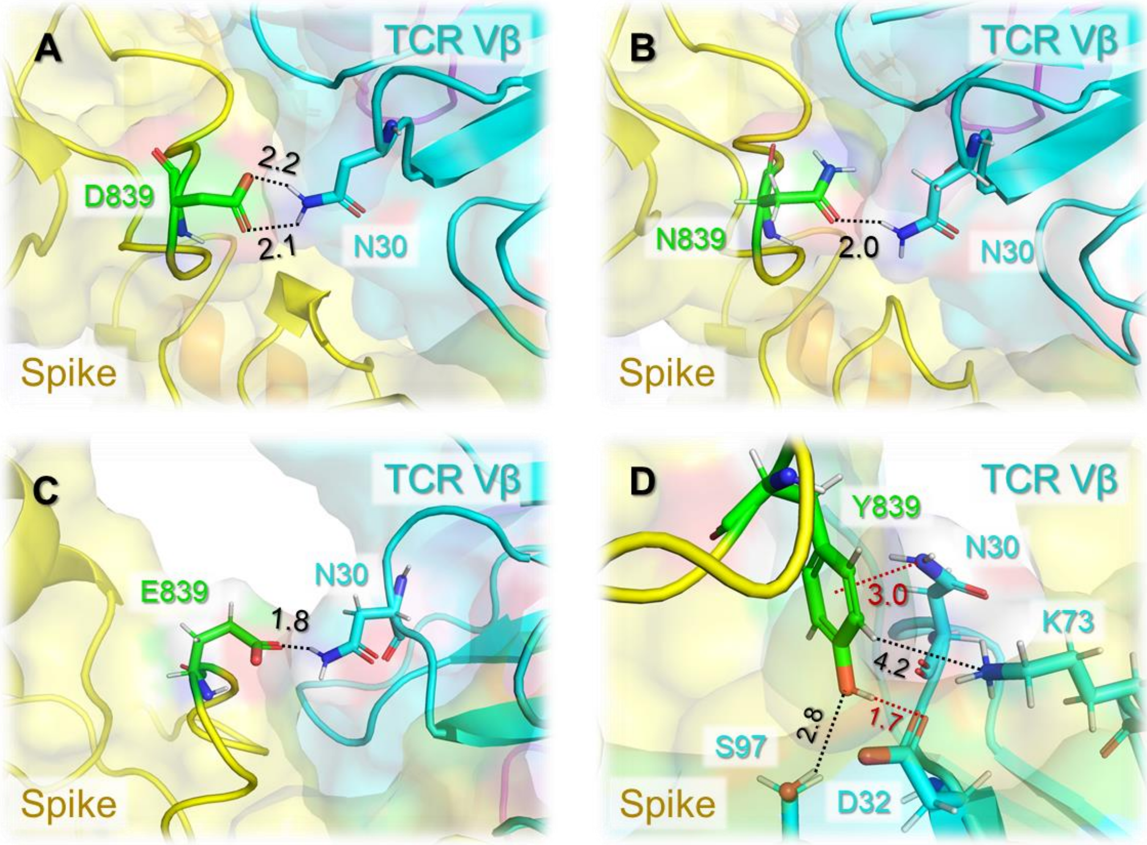

Figure S6: *In silico* mutagenesis analysis of SARS-CoV-2 spike protein residue D839 and its mutation to Y, N, and E. (**A**) A close-up view of the interaction between the wild type residue D839 in the spike and N30 of TCR Vβ. (**B-D**) Results obtained upon mutating D839 to asparagine, glutamic acid, and tyrosine. The spike and TCR Vβ are shown in *yellow* and *cyan*, respectively. The mutation site is highlighted in *green*. Atomic interactions are indicated by *black dashed lines* along with their distances in Ångstroms.

Analysis of NGS immunosequencing data from COVID-19 patients

Blood collection from 38 patients (42 samples) with mild/moderate COVID-19, and 8 patients (24 samples) with severe/hyperinflammatory COVID-19 was performed under institutional review board approval number 2020-039. The patients and controls, and their immune repertoires, were part of a previously published cohort (16). For details of NGS data acquisition, please refer to our earlier work (16). Only productive TRB rearrangements were used and all repertoires were normalized to 20,000 reads. For the analyses, we used R version 3.5.1 for plotting of TRBV and TRBJ gene usage as previously described (17, 18). Differences in principal component analysis were studied by Pillai–Bartlett test of MANOVA. To study TRBJ gene diversity, J genes were extracted if

they were part of rearrangements containing TRBV rearrangements expanded in patients with hyperinflammatory COVID-19. Frequencies of J gene families were summarized per repertoire and plotted separately for each rearrangement. See **Fig S7.**

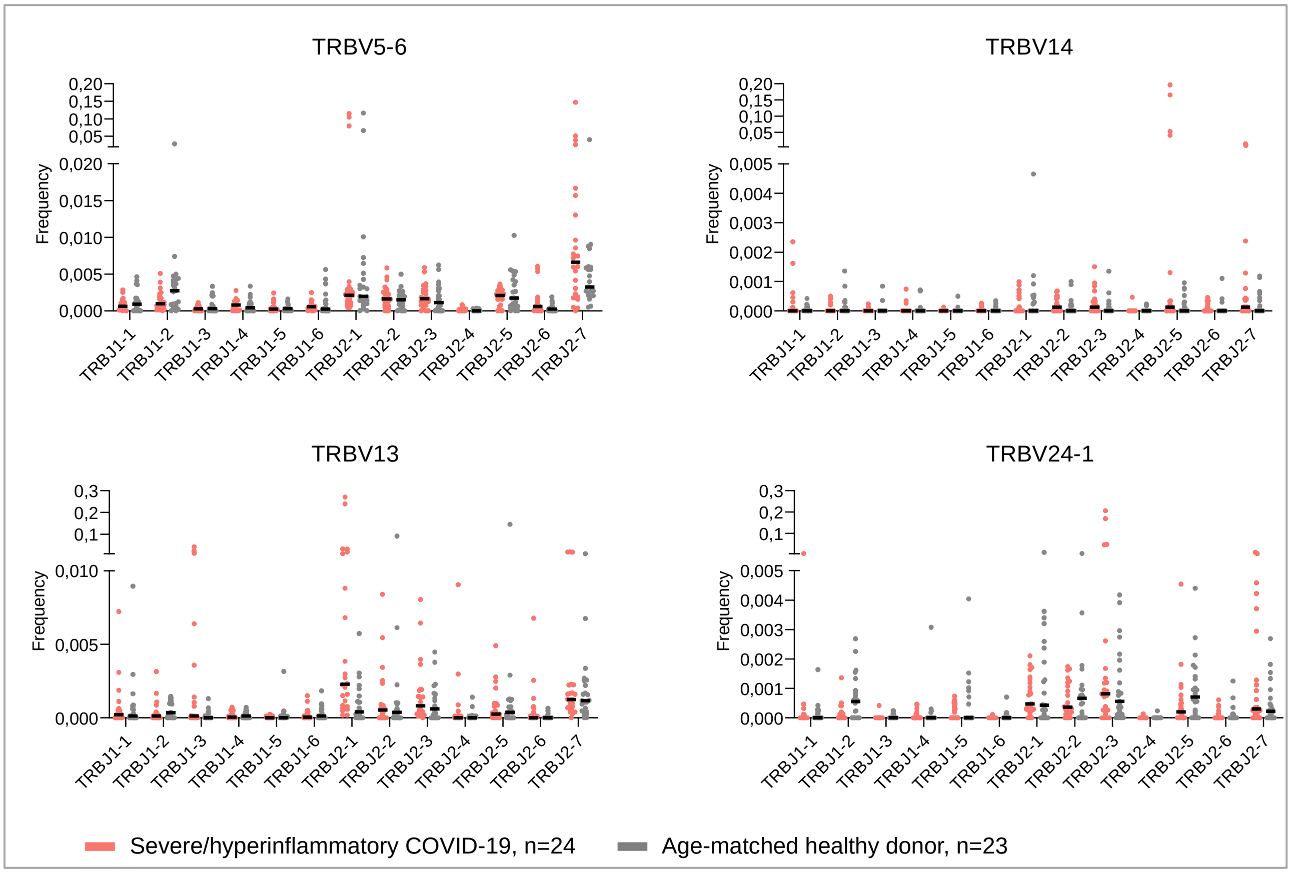

Figure S7: TRBJ-Usage of TRBV genes enriched in hyperinflammatory COVID-19. 24 Repertoires of severe/hyperinflammatory COVID-19 cases versus 23 repertoires of age-matched healthy donors were analyzed. The fraction of individual TRBVJ gene combinations of TRBV5-6, TRBV13, TRBV14 and TRBV24-1 and all 13 different TRBJ genes per repertoire is shown, lines indicating median with interquartile range.

Generation of complexes between SARS-CoV-2 spike, SAg-specific TCRs and MHC II

Four TCR Vβ genes (TRBV5-6, TRBV14, TRBV13 and TRBV24-1) were found to be overrepresented in severe/hyperinflammatory COVID-19 patients (**Fig 6**). We investigated the binding properties of the βTCRs encoded by those genes. To this aim, we extracted from the UniProtKB (23) the amino acid sequences corresponding to these respective genes, used them in FASTA format to search for the corresponding structures, if any, in the Protein Data Bank (PDB) (19), using SwissModel (1). We found structural data in the PDB for TCRVβ chains of three of the genes, TRBV5-6 (UniProt id: A0A599), TRBV14 (A0A5B0), and TRBV24-1 (A0A075B6N3). The respective PDB structures have PDB ids: 6ULR (20), 2ESV (21), and 6EH6 (22). These structures contain both α– and β-chains and

their Vβ domains have 95-100% sequence identity with the Vβ chains encoded by the respective TRVB genes. These PDB structures were used in docking simulations using the software ClusPro (4) to examine their binding properties with respect to the SARS-CoV-2 spike. 30 clusters (obtained upon grouping ~ 700 models) were generated for each of the TCRs complexed with the spike, and in each case there were 3 or more clusters where the TCR was bound to the SAg. **Fig S8** panels **A-C** display representative conformers from these clusters. Panel **D** displays the multiple sequence alignment generated for the TCR Vβ chains (with a few residues of the constant domain succeeding the CDR3). The binding paratopes are indicated by color-coded bars above the alignment. Simulations using the same protocol as the one adopted for generating **Fig S5** predicted that ternary complexes with MHCII were also energetically favorable for all three cases. Panels **E** and **F** illustrate the ternary complexes with MHCII for the TCRs corresponding to TRBV5-6 and TRBV14.

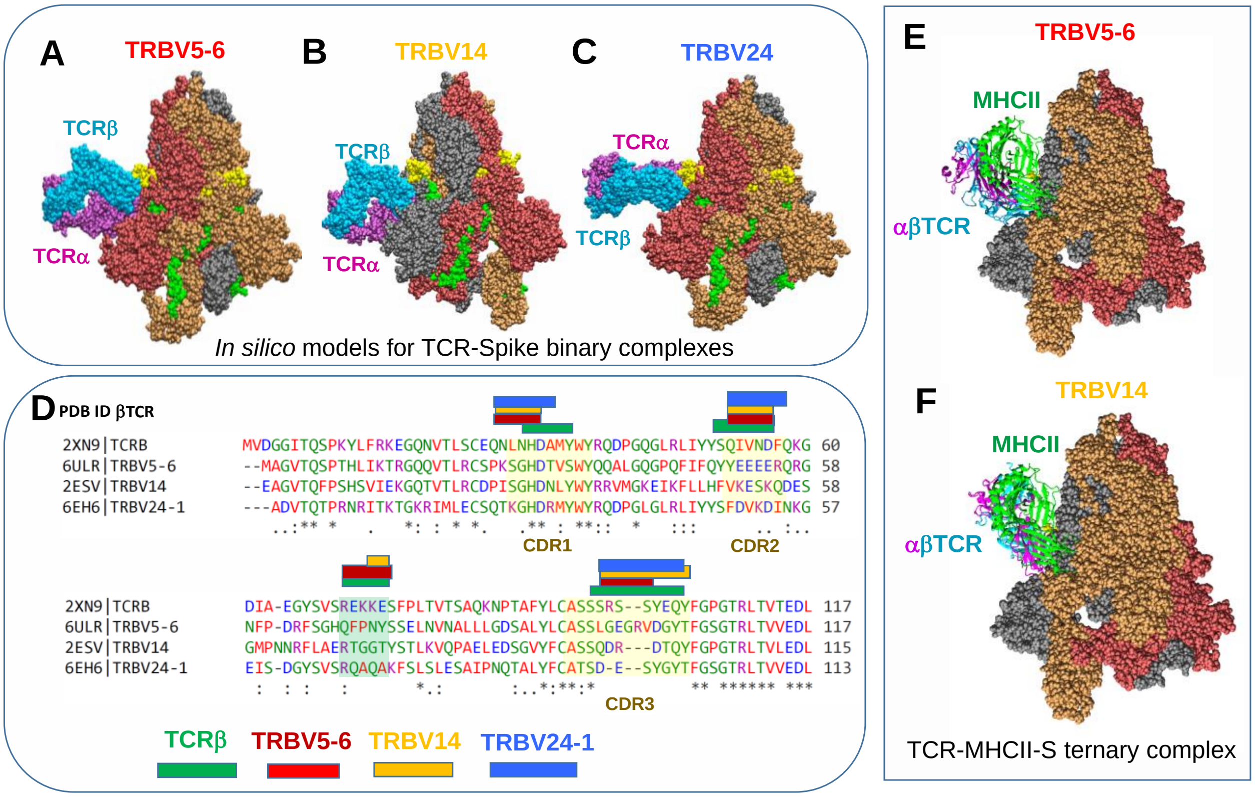

Figure S8: Binding of TCRs (with Vβ chains identical to those overrepresented in the TCR repertoire of patients with severe COVID-19) to the putative SAg site and complexation with MHC II. (A-C) Complexes predicted between SARS-CoV-2 spike SAg-like region and αβTCRs corresponding to the genes (**A**)TCRBV5-6; (**B**)TCRBV14; and (**C**)TCRBV24-1. The spike subunits are colored *dark red*, *orange,* and *gray*; the SAg-like region (E661 to R685) is colored *yellow*; and the neurotoxin motif (299-351) region is *green*. The TCR α- and β-chains are shown in *magenta* and *cyan*, respectively. (**D**) Sequence alignment of the Vβ domain of the TCRs shown in **Fig 1** (TCRB) and in panels **A-C**, generated by Clustal Omega (24). The βTCR paratopes that bind to the SAg-like site on spike are indicated by the color-coded bars, and the CDRs are highlighted in green. Note that there is an additional segment, highlighted in green, which also includes residues making interfacial contacts with the SAg region of the spike, despite its sequence heterogeneity. (**E-F**) Ternary complexes with MHCII predicted for the overrepresented TCRs, illustrated for two cases.

Supplemental Table S1

Binding affinities between the αβTCR and SARS-CoV-2 spike, for the wt and mutant (D839Y/N/E) S glycoproteins

	Aspartic Acid (D)	Tyrosine (Y)	Glutamic Acid (E)	Asparagine (N)
	ΔG (kcal mol^{-1})	ΔG (kcal mol^{-1})	ΔG (kcal mol^{-1})	ΔG (kcal mol^{-1})
**Full complex**	-18.4 ± 0.2	-19.3 ± 0.7	-19.0 ± 1.3	-19.0 ± 0.5
**S subunit - TCR Vβ**	-13.3 ± 0.3	-14.6 ± 0.3	-13.7 ± 0.6	-13.9 ± 0.5

** Binding affinities (ΔG) were obtained at 37 °C using PRODIGY server (14, 15).*

Supplemental References

1. Waterhouse A*, et al.* (2018) SWISS-MODEL: homology modelling of protein structures and complexes. *Nucleic Acids Res* 46(W1):W296-W303.
2. Wrapp D*, et al.* (2020) Cryo-EM structure of the 2019-nCoV spike in the prefusion conformation. *Science* 367(6483):1260-1263.
3. Saline M*, et al.* (2010) The structure of superantigen complexed with TCR and MHC reveals novel insights into superantigenic T cell activation. *Nat. Commun.* 1(1):119.
4. Kozakov D*, et al.* (2017) The ClusPro web server for protein–protein docking. *Nat. Protoc.* 12(2):255.
5. Song W, Gui M, Wang X, & Xiang Y (2018) Cryo-EM structure of the SARS coronavirus spike glycoprotein in complex with its host cell receptor ACE2. *PLoS Pathog.* 14(8):e1007236.
6. Yuan Y*, et al.* (2017) Cryo-EM structures of MERS-CoV and SARS-CoV spike glycoproteins reveal the dynamic receptor binding domains. *Nat Commun* 8:15092.
7. Mateus J*, et al.* (2020) Selective and cross-reactive SARS-CoV-2 T cell epitopes in unexposed humans. *Science*.
8. Li Y*, et al.* (2004) Structure-based preliminary analysis of immunity and virulence of SARS coronavirus. *Viral Immunol.* 17(4):528-534.
9. Zhan SH, Deverman BE, & Chan YA (2020) SARS-CoV-2 is well adapted for humans. What does this mean for re-emergence? *bioRxiv*:2020.2005.2001.073262.
10. Korber B*, et al.* (2020) Spike mutation pipeline reveals the emergence of a more transmissible form of SARS-CoV-2. *bioRxiv*:2020.2004.2029.069054.
11. DeLano WL (2002) Pymol: An open-source molecular graphics tool. *CCP4 Newsletter On Protein Crystallography* 40(1):82-92.
12. Eastman P*, et al.* (2017) OpenMM 7: Rapid development of high performance algorithms for molecular dynamics. *PLoS Comput. Biol.* 13(7):e1005659.
13. Maier JA*, et al.* (2015) ff14SB: Improving the Accuracy of Protein Side Chain and Backbone Parameters from ff99SB. *J. Chem. Theory Comput.* 11(8):3696-3713.

14. Vangone A & Bonvin AM (2015) Contacts-based prediction of binding affinity in protein–protein complexes. *elife* 4:e07454.
15. Xue LC, Rodrigues JP, Kastritis PL, Bonvin AM, & Vangone A (2016) PRODIGY: a web server for predicting the binding affinity of protein–protein complexes. *Bioinformatics* 32(23):3676-3678.
16. Schultheiss C*, et al.* (2020) Next-Generation Sequencing of T and B Cell Receptor Repertoires from COVID-19 Patients Showed Signatures Associated with Severity of Disease. *Immunity* 53:442-455.
17. Simnica D*, et al.* (2019) T cell receptor next-generation sequencing reveals cancer-associated repertoire metrics and reconstitution after chemotherapy in patients with hematological and solid tumors. *Oncoimmunology* 8(11):e1644110.
18. Simnica D*, et al.* (2019) High-Throughput Immunogenetics Reveals a Lack of Physiological T Cell Clusters in Patients With Autoimmune Cytopenias. *Frontiers in Immunology* 10(1897).
19. Berman HM*, et al.* (2000) The protein data bank. *Nucleic acids research* 28(1):235-242.
20. Sim MJW*, et al.* (2020) High-affinity oligoclonal TCRs define effective adoptive T cell therapy targeting mutant KRAS-G12D. *Proceedings of the National Academy of Sciences of the United States of America* 117(23):12826-12835.
21. Hoare HL*, et al.* (2006) Structural basis for a major histocompatibility complex class Ib–restricted T cell response. *Nature Immunology* 7(3):256-264.
22. Holland CJ*, et al.* (2018) In Silico and Structural Analyses Demonstrate That Intrinsic Protein Motions Guide T Cell Receptor Complementarity Determining Region Loop Flexibility. *Frontiers in immunology* 9:674.
23. The UniProt Consortium (2016) UniProt: the universal protein knowledgebase. *Nucleic Acids Research* 45(D1):D158-D169.
24. Sievers F*, et al.* (2011) Fast, scalable generation of high-quality protein multiple sequence alignments using Clustal Omega. *Mol Syst Biol* 7(1):539.
